# The consequence of COVID-19 on the global supply of medical products: Why Indian generics matter for the world?

**DOI:** 10.12688/f1000research.23057.1

**Published:** 2020-04-01

**Authors:** Philippe J Guerin, Sauman Singh-Phulgenda, Nathalie Strub-Wourgaft

**Affiliations:** 1 Infectious Diseases Data Observatory (IDDO), Oxford, UK; 2 Centre for Tropical Medicine and Global Health, Nuffield Department of Clinical Medicine, University of Oxford, Oxford, UK; 3 Drugs for Neglected Diseases initiative (DNDi), Geneva, Switzerland

**Keywords:** generic drugs, COVID-19, India, active pharmaceutical ingredients, manufacturing industry, regulatory agency, medicine access, pharmaceutical industry

## Abstract

While the world is facing the urgency of the COVID-19 pandemic, policymakers must plan for the direct response to the outbreak while minimising its collateral impact. Maintaining the supply chain of pharmaceutical products is not only paramount to cover the immediate medical response but will be fundamental to reducing disruption of the healthcare delivery system, which requires constant medicines, diagnostic tools and vaccines for smooth functioning. In this equation, the role of the Indian pharmaceutical industry will not only be critical to meet the domestic need of over 1.3 billion inhabitants but will equally be important for the rest of the world, including wealthy economies. Preventing a significant disruption of the Indian pharmaceutical supply chain during the outbreak and preparing it for large scale production for COVID-19 therapeutic or preventive medical products will not only help India but will assist the global response to this outbreak.

The COVID-19 pandemic has emerged as an unprecedented global health crisis. Although the extent of the ramifications is still to be established, it is evident that it will have a major impact on global trade in the immediate as well as distant future ^1^. The supply chain of global pharmaceuticals is likely to be interrupted, and the impact on global access to medicine, particularly in low-and middle-income countries (LMICs), will have dramatic consequences.

In 2018–19, India exported nearly $19 billion worth of pharmaceuticals to more than 200 countries, from the highly regulated markets of North America and Europe to countries with limited pharmaceutical industry capacity such as most of sub-Saharan Africa (SSA) ^2^. The Indian Department of Pharmaceuticals reports that formulations and biologicals account for 77% of the total Indian exports and Indian firms provide 20% of the global supply of generics ^3^.

Indian firms thus meet 40% of the generic demand in the US and a quarter of that of Europe ^4, 5^. India accounts for 12% of all manufacturing sites catering to the US market, and 50 Indian firms have a combined abbreviated new drug application (ANDA) market authorization for over 5000 medical products. Similarly, India has 622 sites approved by the European Union and nearly 1700 products with market authorization from the UK Medicines Healthcare Regulatory Agency ^6^. Overall, SSA imports nearly 70% of its pharmaceutical needs ^7^, and India was also the single largest supplier of medicines to Africa in 2018, accounting for a fifth of its pharmaceutical imports ^8^.

Indian firms import almost 70% of their bulk drugs from China, where the production of active pharmaceutical ingredients (APIs) and supply chain logistics have taken a big hit due to the novel coronavirus outbreak ^9^. At the same time, the Indian government has restricted the export of 26 bulk drugs and their formulations ^10^, including several products part of the World Health Organization (WHO) essential medicine list, e.g. antibiotics such as clindamycin, erythromycin, chloramphenicol, one antiretroviral acyclovir, which in total account for 10% of all Indian exports according to Reuters ^11^. India’s production capacity, as well as its export potential, are in other words, already impacting pharmaceutical access in a significant manner.

Furthermore, Indian manufacturers represent 67% (379) of the 563 WHO prequalified pharmaceutical products for a range of conditions such as diarrhoea (1), hepatitis (13), HIV/AIDS (197), influenza (10), malaria (41), neglected tropical diseases (3), reproductive health (21), and tuberculosis (93). A total of 130 of these products are dependent on APIs sourced from China. Besides, 15 Chinese firms are also manufacturing 42 WHO prequalified products ^12^.

Undoubtedly, the most vulnerable to the shock of the destabilization of the Indian industry will be the institutional markets for medicines in LMICs. Indian firms indeed account for over 90% of the antiretroviral (ARV) procurement in LMICs funded through donor procurement ^13^. Two-thirds of the medicines used by important global health players like Médecins Sans Frontières (MSF) to treat HIV, tuberculosis and malaria are generics sourced from India, as well as are treatments for some neglected tropical diseases ^14^. Likewise, 70% of the supply of pentavalent vaccines to UNICEF is dependent on the supply by Indian firms ^15^, while a single eligible Indian firm ‘Serum Institute of India’ supplies the measles vaccine for the Gavi program ^16^.

In the context of the COVID-19 pandemic, global reliance on Indian generics is likely to become a complex international challenge. There are no reliable substitutes for API supplies, nor production capacity available, and more importantly, any country potentially capable of establishing manufacture is likely to focus on national needs and not on export nor development aid.

Mitigation and control of the outbreak of COVID-19 in India are thus of paramount importance not only to India but to the world. Its capacity to import raw materials, manufacture and export medicines will not only determine how the majority of LMICs will be able to respond to the outbreak but will also affect high-income countries. As a major component of the Indian economy, the state of its pharmaceutical industry will also determine the impact of the pandemic on one-fifth of the world’s population. Historically, India has shown commitment at the highest level to ensure the health of millions around the world with a dynamic and resilient pharmaceutical industry. Exceptional measures should be taken in order to support and maintain the operationality of the production plants.

Governments and international organizations who depend on India for their supplies should look beyond their individual demands and support the Indian pharmaceutical supply chain. There is a need to look at contingency plans to assure access to APIs and medicines globally. It is expected that in a few months, diagnostic tools, medicines and vaccines will be approved by medicine regulatory agencies to diagnose, treat and prevent COVID-19 infections. Production at a large scale of those pharmaceutical goods will require the full support of the entire global pharmaceutical industry. Considering the production capability of Indian firms, their engagement will be critical for the rest of the world as well as India to return to some sort of normality post pandemic.

## Data availability

No data are associated with this article.
