# Supplementary material for: The consequence of COVID-19 on the global supply of medical products: Why Indian generics matter for the world?
Source: F1000Res. 2020 Apr 1;9:225. [Version 1] doi: 10.12688/f1000research.23057.1 (PMC7284150; doi:10.12688/f1000research.23057.1)
Supplement: Peer review history

# Peer review: Reviewer response for version 1

**DOI:** 10.5256/f1000research.25454.r63973

**Published:** 2020-06-10

**Authors:** Rory Horner

**Affiliations:** 1 Global Development Institute, University of Manchester, Manchester, UK

The article highlights the major role of India in supplying medicines to the world, relatively well-known to those who specialised in this area of research, but less to the wider world. It is important to have this message at the time of a global pandemic.

 The data on the significance of India’s pharmaceutical imports in Africa – that India contributes one-fifth of its pharmaceutical imports – to some extent underestimates its significance because it is a value number rather than a volume number. More broadly, because of its role supplying generic medicines, which are usually lower-cost, India’s significance is most prominent in volume terms.

 There are two slightly different dynamics that need to be considered vis-à-vis COVID-19 and India’s pharmaceutical industry: 1) the wider impact on the general supply of medical products from India (and maintaining that during the lockdown); 2) India’s role as a supplier of potential COVID-19 treatments (e.g. drugs) or of vaccines to prevent COVID-19 infection.

 The focus of this piece is more on the former, partly reflecting the time the article was written and the major concerns during the initial weeks of the spread of the COVID-19 crisis and India’s lockdown. Most Indian pharmaceutical firms have managed to maintain production during lockdown, albeit at reduced levels. Many of the initial export restrictions placed in India, although subject to considerable media attention beyond India especially, were relatively short-lived. Companies with pre-existing advanced market commitments were quickly issued with exemptions from the export bans.

 The second aspect – related specifically to COVID-19 treatments - is also a crucial aspect of why “Indian generics matter for the world” ^1^ but is only briefly touched on in the latter part of this article. In the just over two months now since this article was originally published, and as Indian companies have managed to maintain some level of production during lockdown, this latter aspect has become especially significant.

 Considerable contestation and international attention has surrounded India’s role in supplying hydroxychloroquine, Remdesevir and a potential vaccine. This aspect is especially important in the need for continued attention to India’s pharmaceutical industry during the pandemic, and is why key stakeholders from elsewhere may need to collaborate with India. For example, Serum Institute is already involved in manufacturing the vaccine candidate being developed at Oxford University which AstraZeneca has a license for, while Gilead has issued licenses to a number of companies in India to produce Remdesevir. A tension between globalisation and nationalism is thus likely to continue to be a key aspect of the Covid-19 response.

Is the topic of the opinion article discussed accurately in the context of the current literature?

Yes

Are arguments sufficiently supported by evidence from the published literature?

Yes

Are all factual statements correct and adequately supported by citations?

Yes

Are the conclusions drawn balanced and justified on the basis of the presented arguments?

Yes

Reviewer Expertise:

Pharmaceutical value chains, India, Africa, industrial and trade policy, globalisation.

I confirm that I have read this submission and believe that I have an appropriate level of expertise to confirm that it is of an acceptable scientific standard.

## References

[1] : The world needs pharmaceuticals from China and India to beat coronavirus. *The Conversation*.2020;(accessed: 25 May 2020):

# Peer review: Reviewer response for version 1

**DOI:** 10.5256/f1000research.25454.r63974

**Published:** 2020-06-09

**Authors:** Yves-Marie Rault Chodankar

**Affiliations:** 1 Centre d’études en sciences sociales sur les mondes africains, américains et asiatiques (CESSMA), Paris, France

The authors draw on secondary data from international institutions and recent research in economics (2016-2020) to highlight the role of India as a major global supplier of pharmaceuticals, particularly to low-and middle-income countries. Writing amid the Covid-19 pandemic, they argue that the disruption of pharmaceutical supply chains due to increases in demand and restrictions on the trade of certain products (e.g. export ban on 26 bulk drugs and formulations manufactured in India) is a major threat to global health and plead for adequate political intervention for the maintenance of a smooth international trade. The article is well documented, written clearly and concisely, and borrows on diversified and reliable data sources to provide figures that are very relevant and significative.

 This article is essential in that it evaluates the importance of India as a global supplier of pharmaceuticals at a time when rigorous data are needed to adequately inform emergency public policies which are too often shaped around political and nationalistic considerations, without looking at the scientific evidence. However, the argument would have likely been stronger if the authors had researched into the products required to fight Covid-19 (e.g. 6-digits level trade data from the UN Comtrade based on the Harmonized System of the World Customs Organization). Although we would have appreciated more attention to the specificities of the pharmaceutical products produced in India, the authors do stress that India’s role is more prevalent in the manufacturing of generic formulations and highlight their dependence on Chinese bulk drugs. In that regard, it would also have been precious to rapidly compare the role of other countries in the global supply of pharmaceuticals like China or Brazil to highlight India's particularities more sharply.

 Although this is not specified in the article, we assume that the main recommendation of the authors on the need to ensure the continuity of international supply chains is meant on a short-term basis, to deal with the immediate aftermath Covid-19 pandemic. We, however, miss here a more specific set of recommendations to achieve this objective, i.e. what is meant by the “full support of the entire global pharmaceutical industry”. It would also have been interesting to discuss whether the current pharmaceutical supply systems, in which countries and firms are increasingly made interdependent, should be modified to ensure more national self-reliance on the long run (e.g. through higher custom duties or incentives to relocate manufacturing units). 

 But all-in-all, this article accurately emphasizes the important role of India in global access to health and can be authoritative as a reference for future research and policies on pharmaceutical supply chains.

Is the topic of the opinion article discussed accurately in the context of the current literature?

Yes

Are arguments sufficiently supported by evidence from the published literature?

Yes

Are all factual statements correct and adequately supported by citations?

Yes

Are the conclusions drawn balanced and justified on the basis of the presented arguments?

Yes

Reviewer Expertise:

global value chains; pharmaceutical market; India

I confirm that I have read this submission and believe that I have an appropriate level of expertise to confirm that it is of an acceptable scientific standard.

# Peer review: Reviewer response for version 1

**DOI:** 10.5256/f1000research.25454.r63976

**Published:** 2020-06-09

**Authors:** Narcyz Ghinea

**Affiliations:** 1 Sydney Health Ethics, Faculty of Medicine and Health, University of Sydney, Sydney, NSW, Australia

This article aims to highlight the importance of the Indian pharmaceutical industry for the global pharmaceutical supply chain and the important role it will play in developing therapeutic goods for COVID-19. I offer the following suggestions for further refinement of the paper.

**Comment 1**: The authors state that India supplies 20% of the worlds global supply of generics. They then state that India provides 40% of supply in the US and 25% in Europe, 70% in sub-Sahara Africa, and 20% in Africa. From these figures it would appear that India provides more than 20% of the global supply. Could these figures be reconciled?

**Comment 2**: While the emphasis in this article is about the critical role the Indian pharma industry plays in the global supply of medicines, one key lesson from the COVID-19 crisis is that there is a strong need to diversify supply chains, rather than falling back into pre-existing "critical" dependencies. This applies both to supply of APIs and manufacturing.   

**Comment 3**: The authors mention 26 bulk drugs account for 10% of all Indian exports. However it is actually 10% of all *pharmaceutical* exports. 

**Comment 4:** It makes more sense to say Indian pharmaceutical companies "manufacture" or "export" 67% of WHO prequalified products, rather than "represent 67%" of these products.

**Comment 5**: In the last paragraph the authors state "Governments and international organizations who depend on India for their supplies should look beyond their individual demands and support the Indian pharmaceutical supply chain". I am not sure what demands are being referred to - could the authors elaborate?

**Comment 6**: I am not sure whether the fact that India accounts for 20% of the current global supply of generics translates into their engagement being "critical for the rest of the world['s]" future response to COVID-19 as the authors conclude. Other countries clearly have capacity to produce generics (and are currently producing 80% worth), so how the race to fulfil demand plays out is yet to be seen. 

**Comment 7**: Given the clear reliance by what the authors call "institutional markets" on the Indian industry, perhaps the focus of the paper should be on these markets? This would be more convincing to me. For instance, in the conclusion the authors could note that since a majority of donor procured medicines used to treat HIV, tuberculosis, malaria and other tropical diseases in LMICs countries are supplied by India, it makes sense that India will play a *critical* role in supporting any COVID-19 response in these countries.

Is the topic of the opinion article discussed accurately in the context of the current literature?

Yes

Are arguments sufficiently supported by evidence from the published literature?

Yes

Are all factual statements correct and adequately supported by citations?

Yes

Are the conclusions drawn balanced and justified on the basis of the presented arguments?

Partly

Reviewer Expertise:

Pharmaceutical policy.

I confirm that I have read this submission and believe that I have an appropriate level of expertise to confirm that it is of an acceptable scientific standard, however I have significant reservations, as outlined above.

# Peer review: Reviewer response for version 1

**DOI:** 10.5256/f1000research.25454.r61902

**Published:** 2020-04-07

**Authors:** Agnès Saint-Raymond

**Affiliations:** 1 International Affairs Division, European Medicines Agency (EMA), Amsterdam, The Netherlands

The opinion article is well written and extremely documented, and the statements well supported by figures and data. It aims at giving a voice to Low-and-Middle Income Countries (LMICs) which are both affected by COVID-19, but also are going to be hit hard by the supply chain disruption in India. 

 India and the rest of the world rely heavily on drugs, mostly generics, and vaccines manufactured in India. India is the main manufacturer of vaccines for UNICEF and of WHO prequalified medicines, i.e. those needed to respond to the major disease burden of LMICs. India itself depends on China, which produces about 80% of its APIs and has already been severely affected by COVID-19 as of December 2019. The pandemic is now affecting India. India is reacting like many countries by protecting its own and putting an export ban on many essential medicines. The disruption of the supply chain will impact LMICs, as they have no or very limited capacity and capability to manufacture and certainly to compensate swiftly. They cannot compete to procure drugs if prices are increased.

 There is a need for global cooperation and rapid action to maintain the supply chain from India towards LMICs.

**Is the topic of the opinion article discussed accurately in the context of the current literature?**

 Yes the article provides extensive evidence of what is the supply chain and its interdependencies. It clearly identifies the high level of dependence on India (and China) by LMICs. 

**Are all factual statements correct and adequately supported by citations?**

 The authors highlight the complexity of the relation between China and India and the high dependency on Indian manufacturers for essential medicines for LMICs. All references are recent (2016-2020).

**Are arguments sufficiently supported by evidence from the published literature?**

 In order to mitigate the risk of supply disruption, it would be good to recall some of the factors contributing to the world’s dependency on India and China in LMICs as well as HMICs (for example 80% of EU medicines are manufactured in either India or China). Such dependency has increased dramatically over the years, due to both countries’ investments in pharmaceutical business and competitiveness, resulting in much lower costs of medicines. Low cost was always hailed as a success, as it allows accessible and affordable medicines in LMICs. What was overlooked was the dependency and its potential consequences in case of severe disruptions of the whole of India, China or both, which could have been predicted from any viral pandemic. Causes are not the focus of this opinion, but this vulnerability will remain. The reasons for low costs would require a specific analysis (e.g. competitiveness, patent laws, state subsidies, economy of scale, social and environmental aspects) and would justify a separate article.

**Are the conclusions drawn balanced and justified on the basis of the presented arguments?**

 The conclusion is very clear and in line with the arguments. It is however quite general, stopping short of making proposals on how to mitigate the risks of supply chain disruption for LMICs. For example, to ensure equitable supply of limited production, which neutral body or international organisation(s), should or could take the lead? When a large proportion of the world including India is in lock-down, the countries’ economies are hard hit or even crashing, without a vaccine immediately available to limit the spread, it will take more than goodwill to reverse the national priority or protection approaches (in India and elsewhere), and to ensure international collaboration towards equitable supply. Some ideas should be added to the conclusion.

Is the topic of the opinion article discussed accurately in the context of the current literature?

Yes

Are arguments sufficiently supported by evidence from the published literature?

Yes

Are all factual statements correct and adequately supported by citations?

Yes

Are the conclusions drawn balanced and justified on the basis of the presented arguments?

Yes

Reviewer Expertise:

International regulatory Affairs. Clinical trials, Paediatrics and Orphan drugs

I confirm that I have read this submission and believe that I have an appropriate level of expertise to confirm that it is of an acceptable scientific standard.
